# Supplementary material for: Skeletal Muscle MicroRNA Patterns in Response to a Single Bout of Exercise in Females: Biomarkers for Subsequent Training Adaptation?
Source: Biomolecules. 2023 May 24;13(6):884. doi: 10.3390/biom13060884 (PMC10296578; doi:10.3390/biom13060884)
Supplement: Supplementary file 1 [file biomolecules-13-00884-s001.zip › biomolecules-2356723-supplementary.pdf]

miR	pre (log(2))	post (log(2))	fold change	p	FDR p
hsa-miR-5189-3p	9.16	0.8	-356.42	***	***
hsa-miR-2115-5p	7.54	0.65	-134.84	***	***
hsa-miR-6808-3p	6.13	0.73	-43.36	***	***
hsa-miR-6806-3p	4.77	0.63	-19.12	***	***
hsa-miR-4330	4.24	0.56	-16.64	***	***
hsa-miR-101-5p	4.1	0.65	-11.54	***	***
hsa-miR-4635	3.72	0.7	-10.8	***	***
hsa-miR-30e-3p	4.97	2.11	-10.49	***	***
hsa-miR-152-3p	6.71	4.44	-7.82	***	**
hsa-miR-199a-5p	4.66	2.12	-7.69	**	0.068
hsa-miR-499a-5p	3.63	1.64	-7.61	***	*
hsa-miR-18a-5p	5.67	3.74	-7.39	**	0.0808
hsa-miR-30a-3p	7.29	4.83	-7.15	***	***
hsa-miR-660-5p	3.6	1.23	-7.06	**	*
hsa-miR-29c-3p	3.14	1.29	-6.91	*	0.1392
hsa-miR-30e-5p	4.86	2.35	-6.87	***	**
hsa-miR-497-5p	3.12	1.12	-6.46	**	0.07
hsa-miR-128-3p	6.07	3.92	-6.36	***	**
hsa-miR-489-3p	4.5	2.73	-6.19	*	0.1814
hsa-miR-4663	2.83	0.79	-5.85	***	*
hsa-miR-199a-3p	7.62	5.42	-5.80	***	**
hsa-miR-199b-3p	7.62	5.42	-5.80	***	**
hsa-miR-192-5p	3.40	1.97	-5.36	**	*
hsa-miR-363-3p	4.15	2.115	-5.36	**	0.1016
hsa-miR-19b-3p	7.93	5.71	-5.33	***	**
hsa-miR-22-5p	3.53	1.90	-5.26	**	0.0654
hsa-miR-15a-5p	5.86	3.73	-5.12	***	*
hsa-miR-10a-5p	3.5	1.75	-4.93	**	*
hsa-miR-1	10.22	8.12	-4.91	***	***
hsa-miR-106b-5p	7.91	6.45	-4.77	*	0.1182
hsa-miR-3175	2.16	2.04	-4.74	0.9208	0.9764
hsa-miR-362-5p	3.75	2.68	-4.69	0.1397	0.5140
hsa-miR-27a-3p	8.21	6.22	-4.64	***	***
hsa-miR-4306	3.06	2.04	-4.57	0.1692	0.5486
hsa-miR-133a-5p	6.97	4.98	-4.54	***	***
hsa-miR-8089	3.13	4.99	4.60	***	*
hsa-miR-3188	2.75	4.48	4.62	**	*
hsa-miR-939-5p	1.76	3.67	4.65	***	**
hsa-miR-197-5p	3.75	5.725	4.85	***	**
hsa-miR-6124	3.57	5.475	4.92	***	*
hsa-miR-1229-5p	2.66	4.60	4.97	***	***
hsa-miR-7151-3p	1.05	3.3	5.08	***	***
hsa-miR-2110	2.77	4.72	5.57	**	*
hsa-miR-6848-5p	2.48	4.69	5.60	***	**
hsa-miR-6831-5p	4.78	6.53	5.64	**	0.0872

hsa-miR-7109-5p	3.60	5.70	5.66	**	*
hsa-miR-4667-5p	2.69	4.68	5.74	**	0.068
hsa-miR-7162-3p	1.53	3.93	6.15	***	*
hsa-miR-132-3p	1.86	4.25	6.46	***	**
hsa-miR-155-5p	0.97	3.605	7.32	***	***
hsa-miR-1226-5p	1.26	4.25	8.32	***	***
hsa-miR-7641	4.055	5.46	17.34	0.1420	0.5157
hsa-miR-6846-5p	1.53	6.12	24.37	***	***
hsa-miR-4743-5p	2.42	7.98	47.98	***	***
hsa-miR-4505	6.08	11.44	49.64	***	***

Suppl. Tab.1. miRs found to be regulated 4.5-fold or more in microarray analysis (n = 6). Values are log(2)-transformed data, as calculated by the program Transcriptome Analysis Console (TAC). P values and FDR (false discovery rate-corrected) p values are given for each miR. Differential expression of miRs labelled in red was subsequently verified by qPCR analysis. *: $p \leq 0.05$; **: $p \leq 0.01$; ***: $p \leq 0.001$.

training #1	$\dot{V}O_2$max. T0 [mL/kgxmin]	$\dot{V}O_2$max. T1 [mL/kgxmin]	$\Delta\dot{V}O_2$max. T0/T1 [mL/kgxmin]	training #2	$\dot{V}O_2$max. T2 [mL/kgxmin]	$\Delta\dot{V}O_2$max. T0/T2 [mL/kgxmin]	$\Delta\dot{V}O_2$max. T1/T2 [mL/kgxmin]
**all participants (n=29)**	29.98 ±3.22	33.92 ±4.02	3.8 ±2.45	**all participants (n=23)**	34.89 ±4.1	4.98 ±3.08	0.91 ±2.78
**HIIT-MICT (n=13)**	30.84 ±2.74	36.07 ±2.61	5.23 ±1.69	**HIIT-MICT (n=11)**	36.2 ±3.55	4.81 ±3.04	-0.51 ±2.9
**MICT-HIIT (n=16)**	29.28 ±3.4	31.91 ±3.7	2.63 ±2.36	**MICT-HIIT (n=12)**	33.69 ±3.81	4.92 ±3.11	2.22 ±1.88

Suppl. Tab. 2. $\dot{V}O_2$max data (means and standard deviations) of all subjects included in qPCR analysis. The first six weeks of training (training #1) were completed by n=29 female participants, the second block (training #2) only by n=23, due to COVID19-associated restrictions. “HIIT-MICT” corresponds to subjects randomized to starting with HIIT (training #1) and then switching to MICT (training #2), “MICT-HIIT” to the group that completed the training sessions in reverse order.

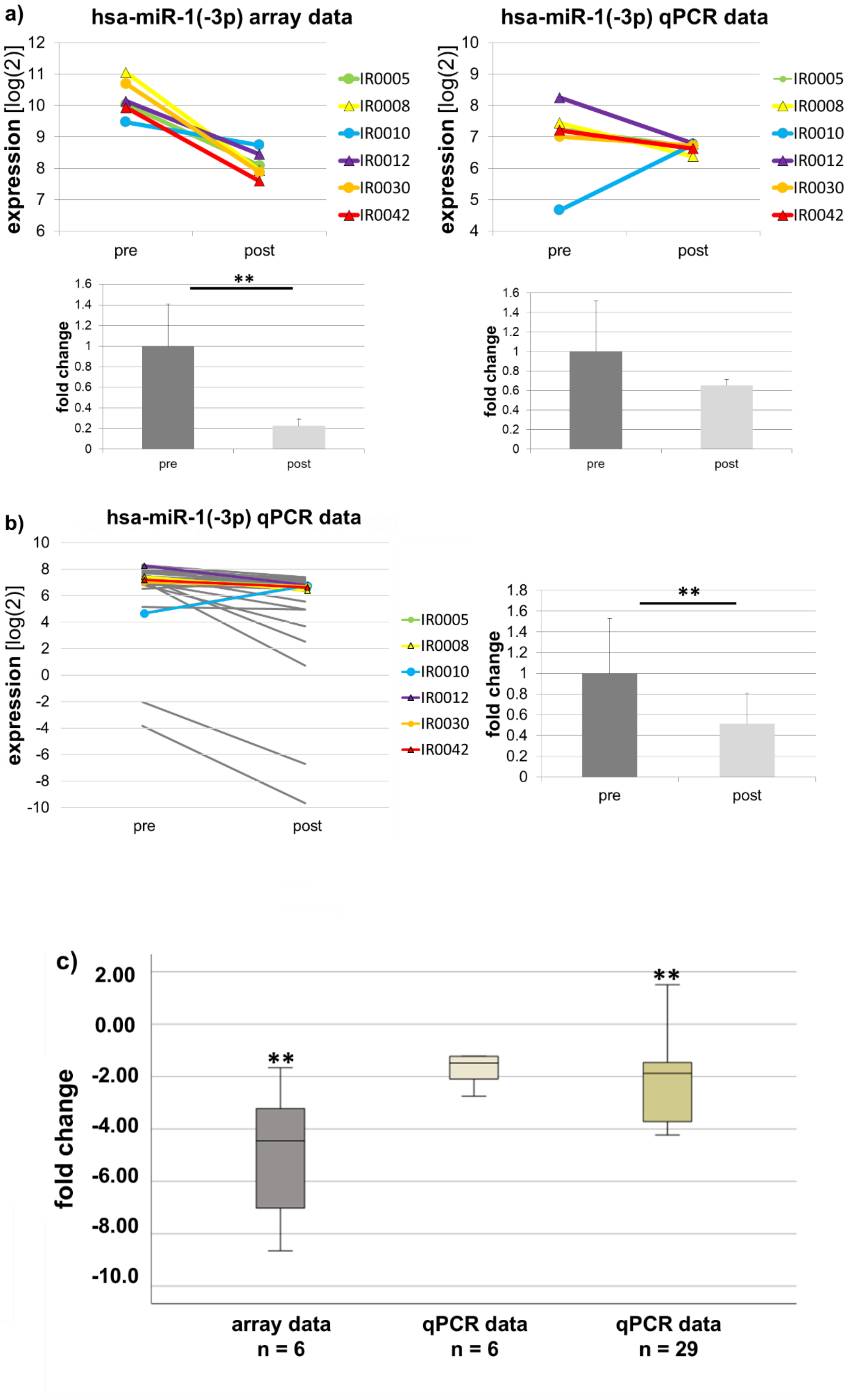
a)
hsa-miR-1(-3p) array data
expression [log(2)]
12
11
10
9
8
7
6
pre
post
IR0005
IR0008
IR0010
IR0012
IR0030
IR0042
hsa-miR-1(-3p) qPCR data
expression [log(2)]
10
9
8
7
6
5
4
pre
post
IR0005
IR0008
IR0010
IR0012
IR0030
IR0042
fold change
1.6
1.4
1.2
1
0.8
0.6
0.4
0.2
0
**
pre
post
fold change
1.6
1.4
1.2
1
0.8
0.6
0.4
0.2
0
pre
post
b)
hsa-miR-1(-3p) qPCR data
expression [log(2)]
10
8
6
4
2
0
-2
-4
-6
-8
-10
pre
post
IR0005
IR0008
IR0010
IR0012
IR0030
IR0042
fold change
1.8
1.6
1.4
1.2
1
0.8
0.6
0.4
0.2
0
**
pre
post
c)
fold change
2.00
0.00
-2.00
-4.00
-6.00
-8.00
-10.0
**
**
array data
n = 6
qPCR data
n = 6
qPCR data
n = 29

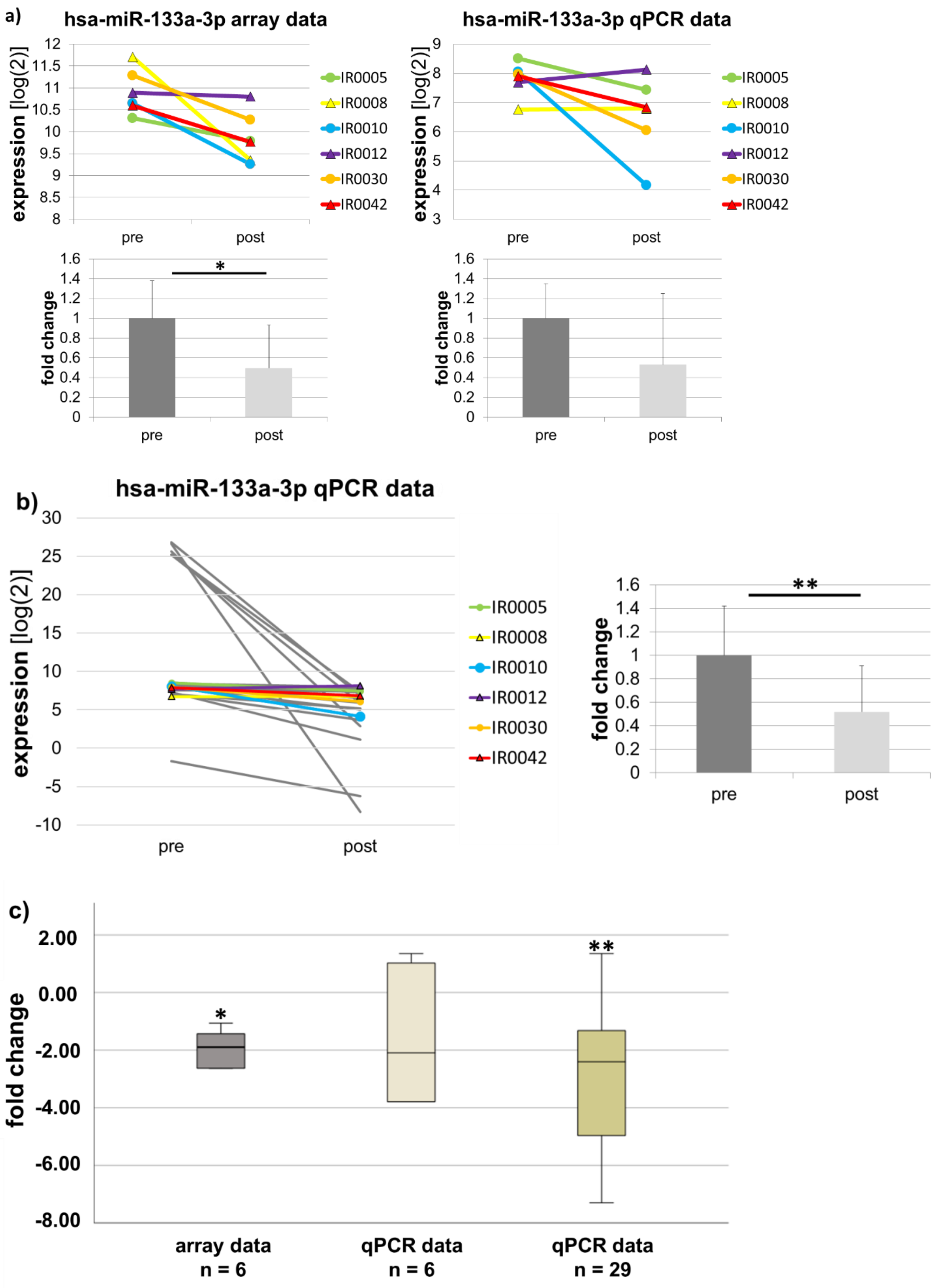
a)
hsa-miR-133a-3p array data
hsa-miR-133a-3p qPCR data
expression [log(2)]
fold change
pre
post
*
IR0005
IR0008
IR0010
IR0012
IR0030
IR0042
b)
hsa-miR-133a-3p qPCR data
expression [log(2)]
fold change
pre
post
**
c)
fold change
array data
n = 6
qPCR data
n = 6
qPCR data
n = 29

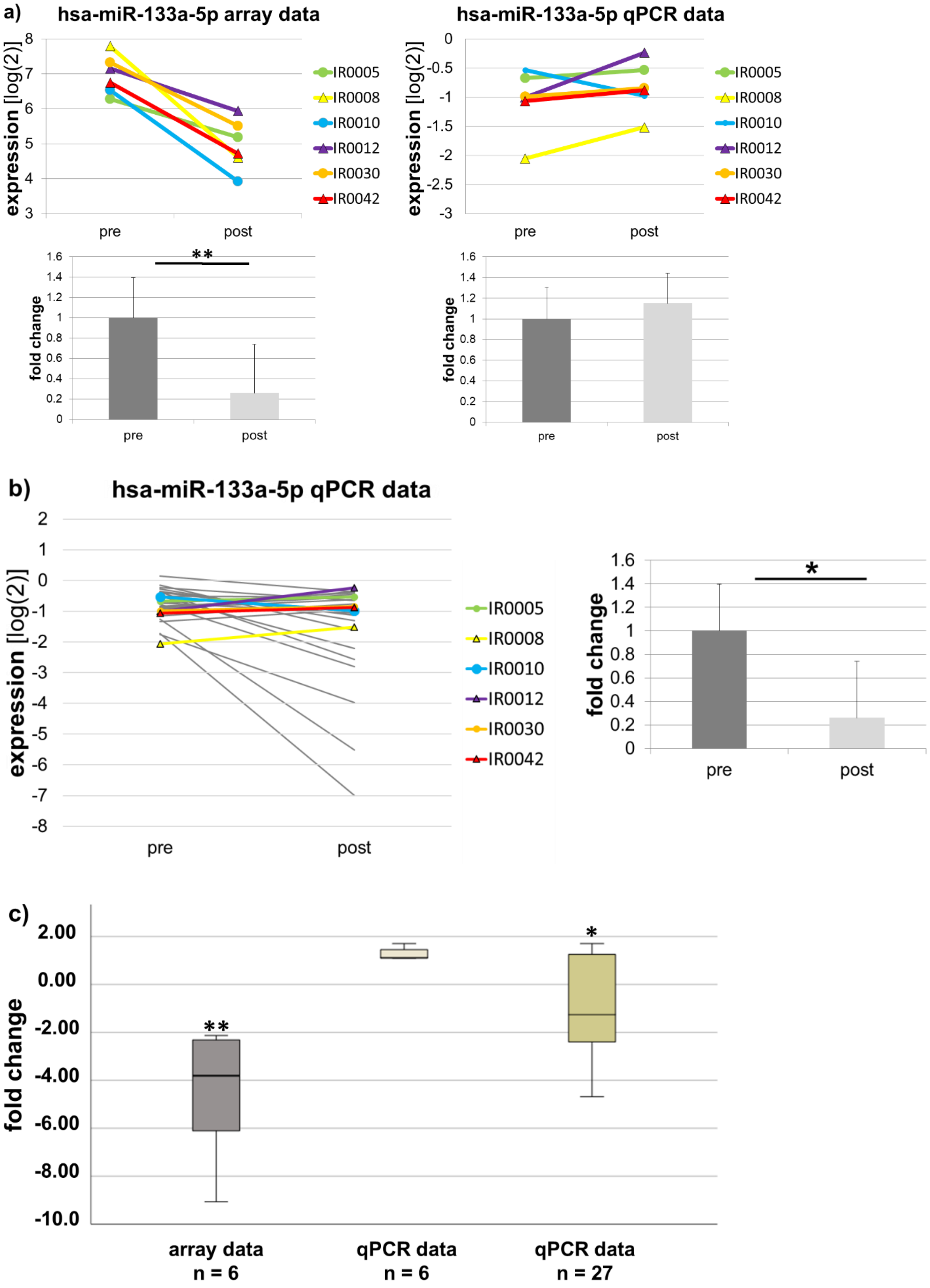

a)
hsa-miR-133a-5p array data
expression [log(2)]
pre
post
IR0005
IR0008
IR0010
IR0012
IR0030
IR0042
hsa-miR-133a-5p qPCR data
fold change
**
b)
hsa-miR-133a-5p qPCR data
*
c)
array data
n = 6
qPCR data
n = 6
qPCR data
n = 27

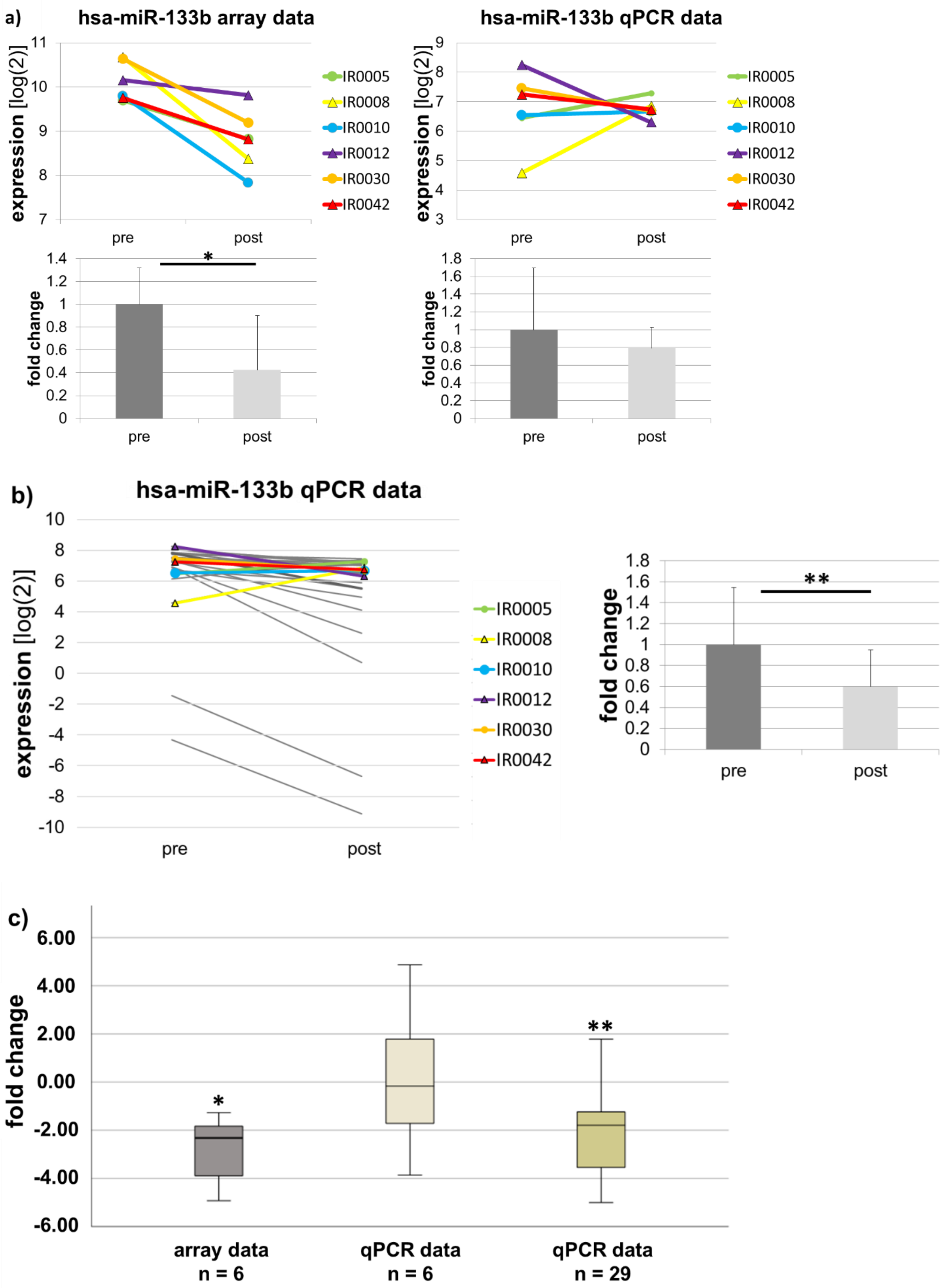

a)
hsa-miR-133b array data
hsa-miR-133b qPCR data
expression [log(2)]
IR0005
IR0008
IR0010
IR0012
IR0030
IR0042
pre
post
fold change
*
b)
hsa-miR-133b qPCR data
expression [log(2)]
fold change
**
c)
fold change
*
**
array data
n = 6
qPCR data
n = 6
qPCR data
n = 29

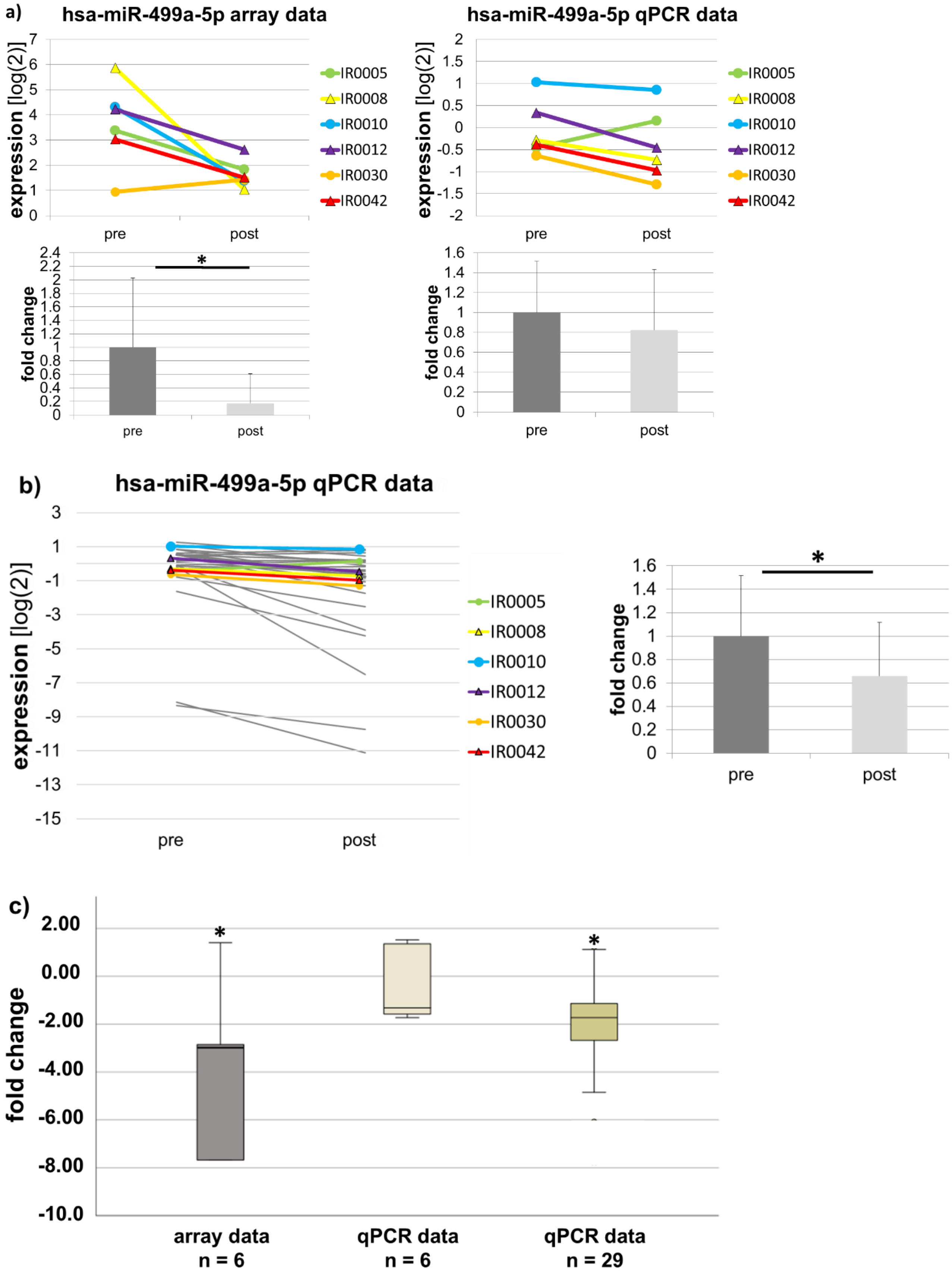

a)
hsa-miR-499a-5p array data
expression [log(2)]
7
6
5
4
3
2
1
0
pre
post
IR0005
IR0008
IR0010
IR0012
IR0030
IR0042
hsa-miR-499a-5p qPCR data
2
1.5
1
0.5
0
-0.5
-1
-1.5
-2
fold change
*
2.4
2.2
2
1.8
1.6
1.4
1.2
1
0.8
0.6
0.4
0.2
0
b)
hsa-miR-499a-5p qPCR data
3
-1
-3
-5
-7
-9
-11
-13
-15
c)
2.00
0.00
-2.00
-4.00
-6.00
-8.00
-10.0
array data
n = 6
qPCR data
n = 6
qPCR data
n = 29

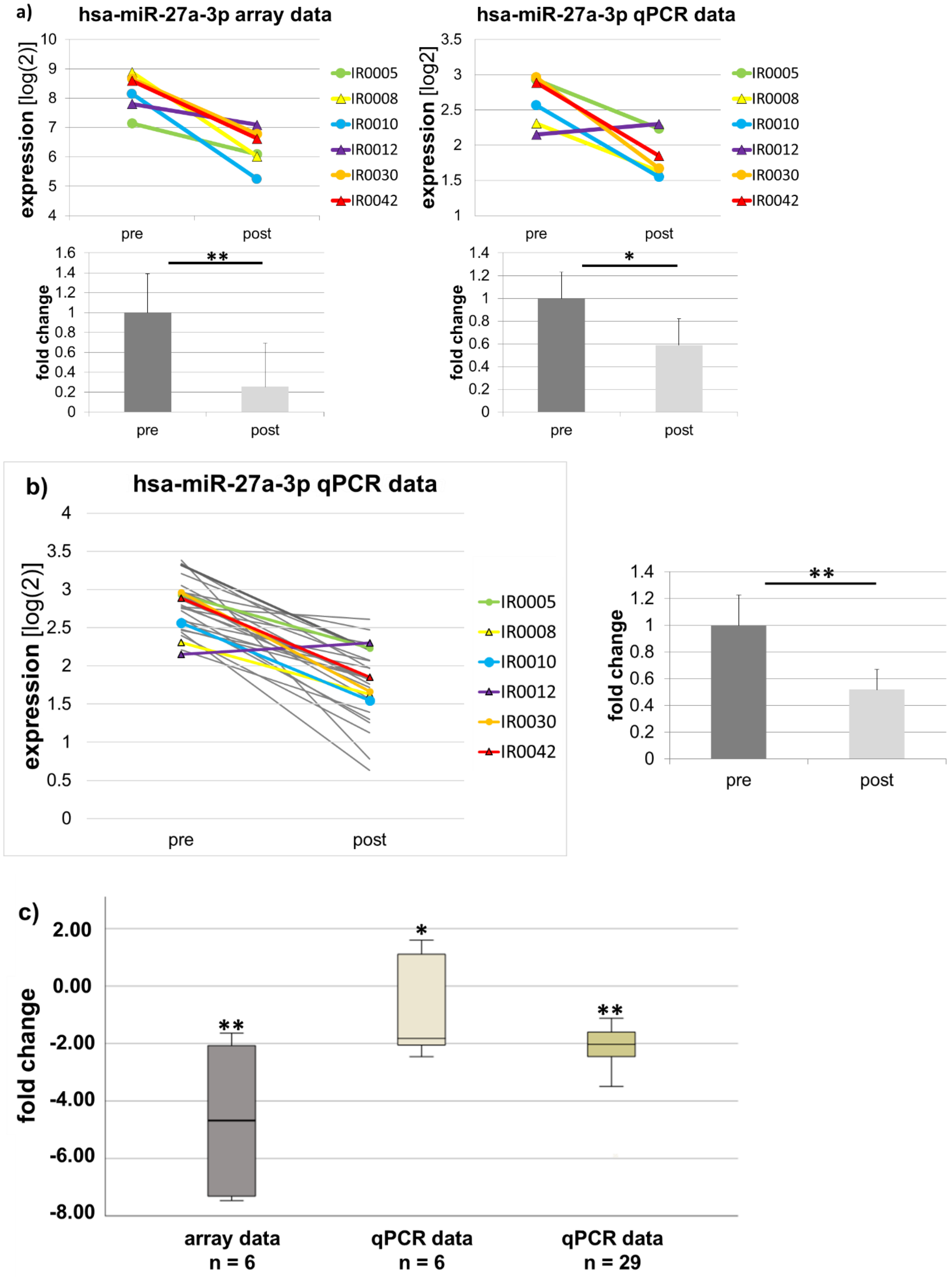
a)
hsa-miR-27a-3p array data
expression [log(2)]
pre
post
IR0005
IR0008
IR0010
IR0012
IR0030
IR0042
fold change
**
hsa-miR-27a-3p qPCR data
expression [log2]
*
b)
hsa-miR-27a-3p qPCR data
expression [log(2)]
**
c)
fold change
array data
n = 6
qPCR data
n = 6
qPCR data
n = 29

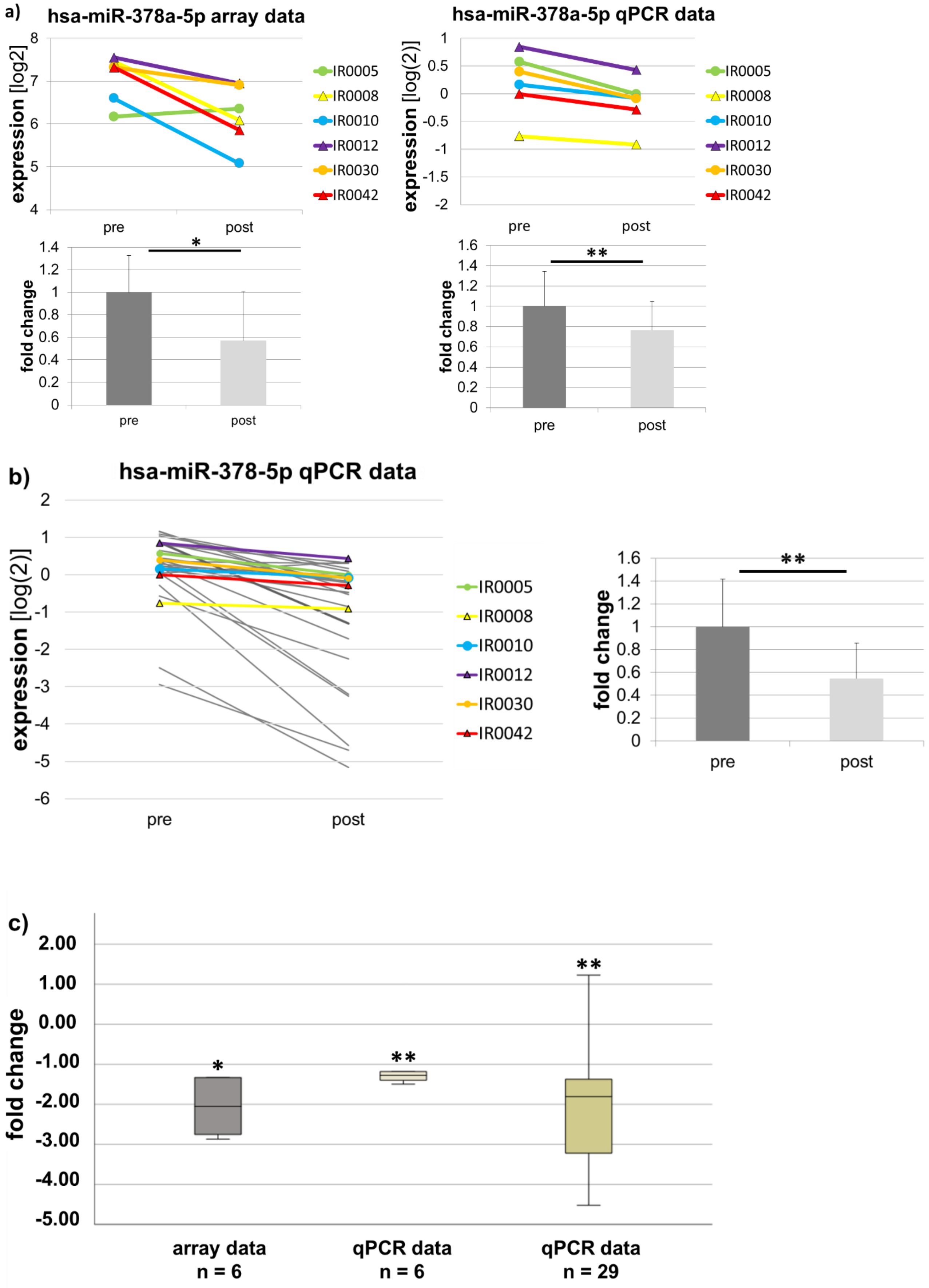

a)
hsa-miR-378a-5p array data
expression [log2]
pre
post
IR0005
IR0008
IR0010
IR0012
IR0030
IR0042
fold change
*
hsa-miR-378a-5p qPCR data
expression [log(2)]
**
b)
hsa-miR-378-5p qPCR data
expression [log(2)]
fold change
**
c)
fold change
array data
n = 6
qPCR data
n = 6
qPCR data
n = 29

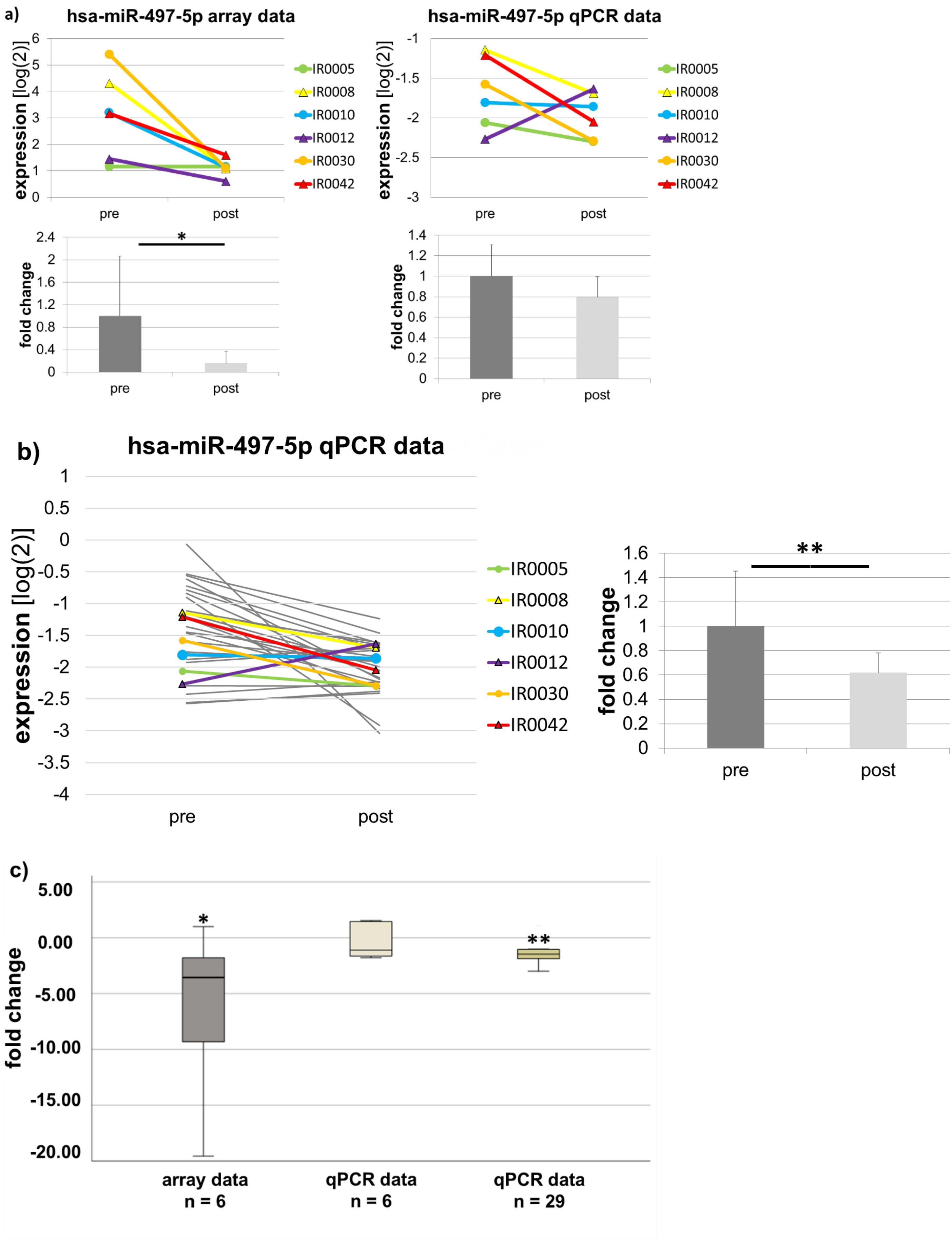
a)
hsa-miR-497-5p array data
hsa-miR-497-5p qPCR data
expression [log(2)]
fold change
pre
post
*
IR0005
IR0008
IR0010
IR0012
IR0030
IR0042
b)
hsa-miR-497-5p qPCR data
**
c)
array data
n = 6
qPCR data
n = 6
qPCR data
n = 29

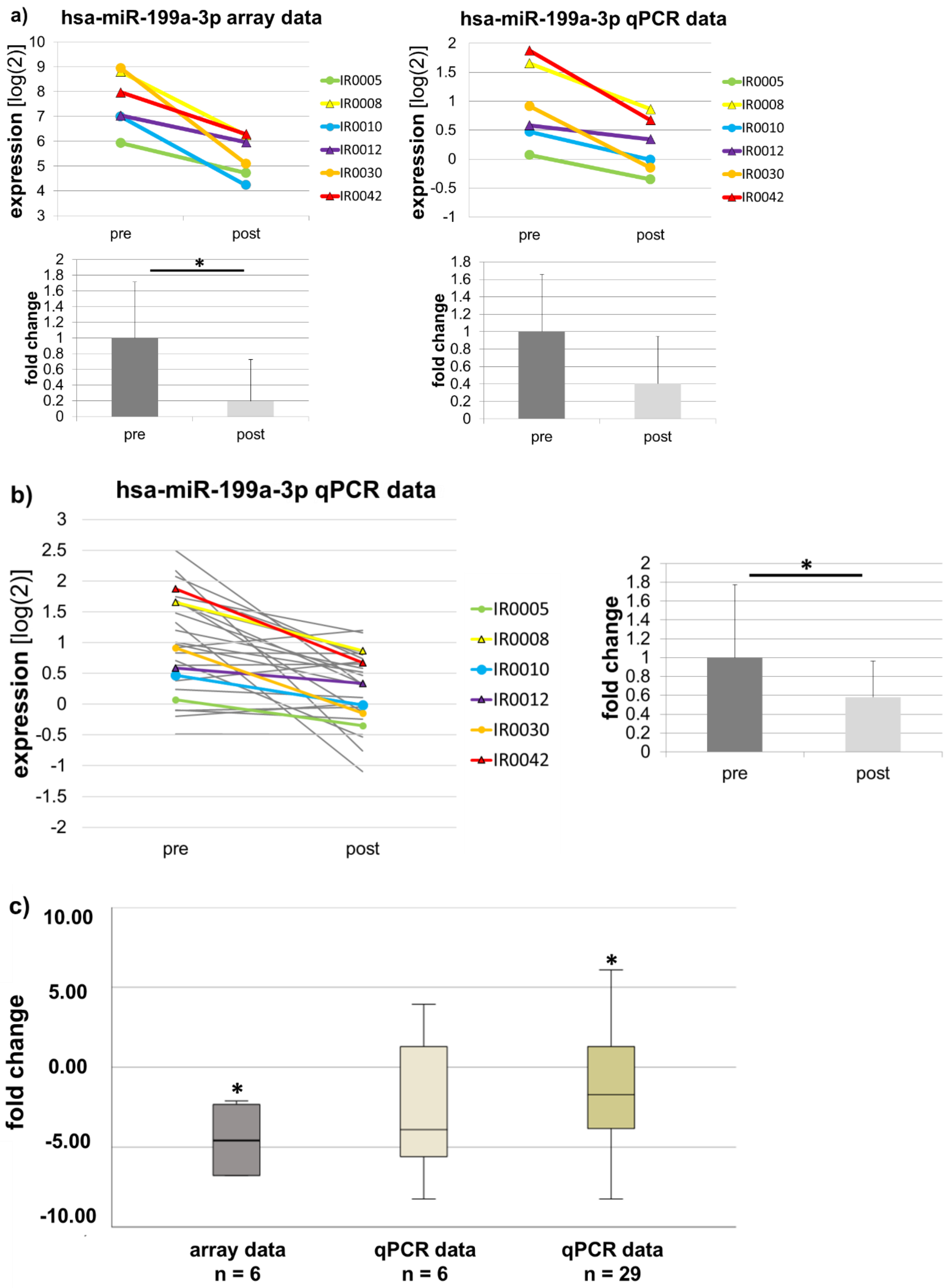
a)
hsa-miR-199a-3p array data
hsa-miR-199a-3p qPCR data
expression [log(2)]
fold change
pre
post
IR0005
IR0008
IR0010
IR0012
IR0030
IR0042
*
b)
hsa-miR-199a-3p qPCR data
expression [log(2)]
fold change
pre
post
*
c)
fold change
array data
n = 6
qPCR data
n = 6
qPCR data
n = 29
*

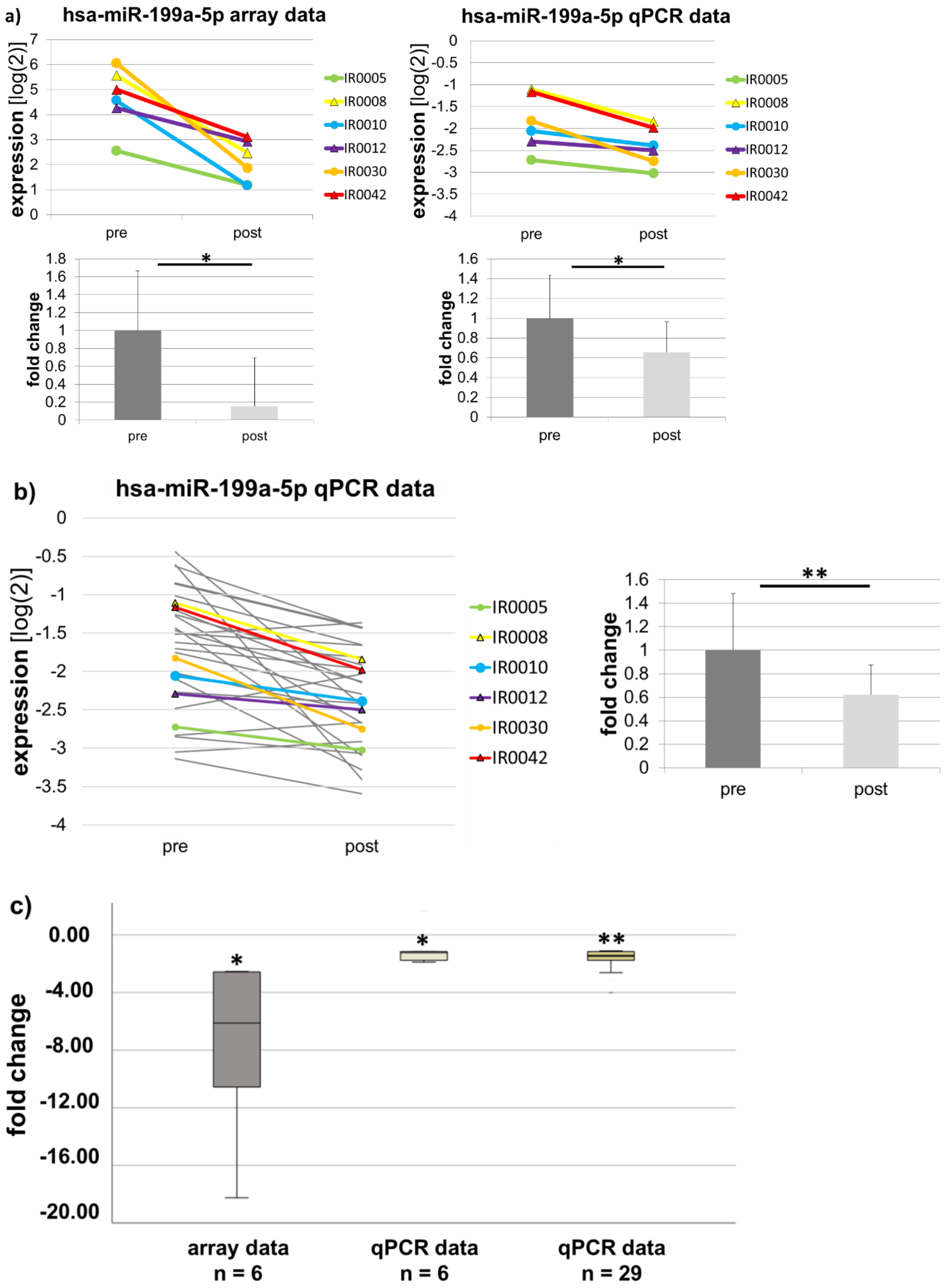

a)
hsa-miR-199a-5p array data
expression [log(2)]
7
6
5
4
3
2
1
0
pre
post
IR0005
IR0008
IR0010
IR0012
IR0030
IR0042
hsa-miR-199a-5p qPCR data
0
-0.5
-1
-1.5
-2
-2.5
-3
-3.5
-4
fold change
1.8
1.6
1.4
1.2
1
0.8
0.6
0.4
0.2
*
b)
hsa-miR-199a-5p qPCR data
**
c)
0.00
-4.00
-8.00
-12.00
-16.00
-20.00
array data
n = 6
qPCR data
n = 6
qPCR data
n = 29

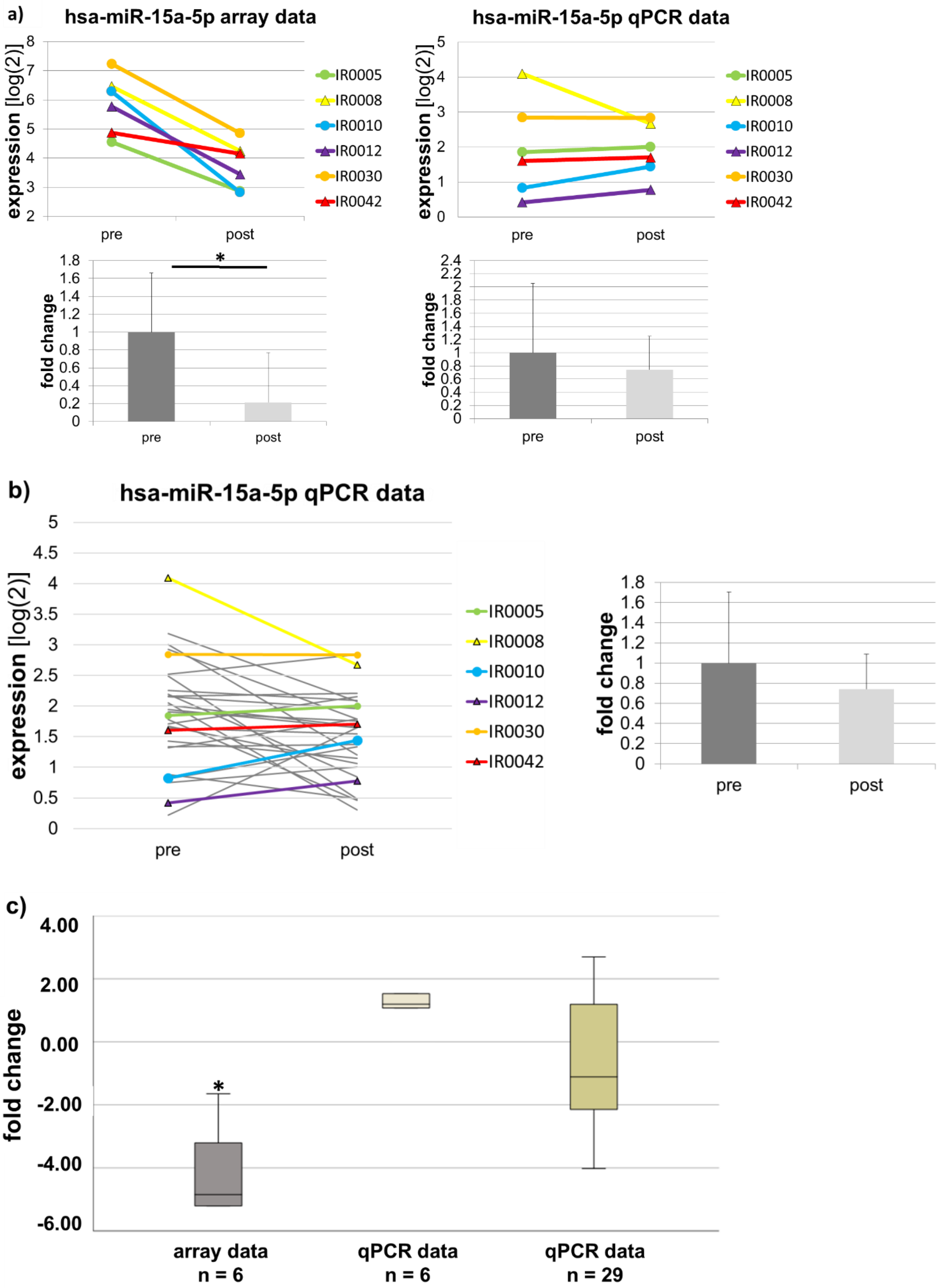
a)
hsa-miR-15a-5p array data
expression [log(2)]
8
7
6
5
4
3
2
pre
post
IR0005
IR0008
IR0010
IR0012
IR0030
IR0042
hsa-miR-15a-5p qPCR data
expression [log(2)]
5
4
3
2
1
0
pre
post
IR0005
IR0008
IR0010
IR0012
IR0030
IR0042
fold change
1.8
1.6
1.4
1.2
1
0.8
0.6
0.4
0.2
0
*
pre
post
fold change
2.4
2.2
2
1.8
1.6
1.4
1.2
1
0.8
0.6
0.4
0.2
0
pre
post
b)
hsa-miR-15a-5p qPCR data
expression [log(2)]
5
4.5
4
3.5
3
2.5
2
1.5
1
0.5
0
pre
post
IR0005
IR0008
IR0010
IR0012
IR0030
IR0042
fold change
1.8
1.6
1.4
1.2
1
0.8
0.6
0.4
0.2
0
pre
post
c)
fold change
4.00
2.00
0.00
-2.00
-4.00
-6.00
*
array data
n = 6
qPCR data
n = 6
qPCR data
n = 29

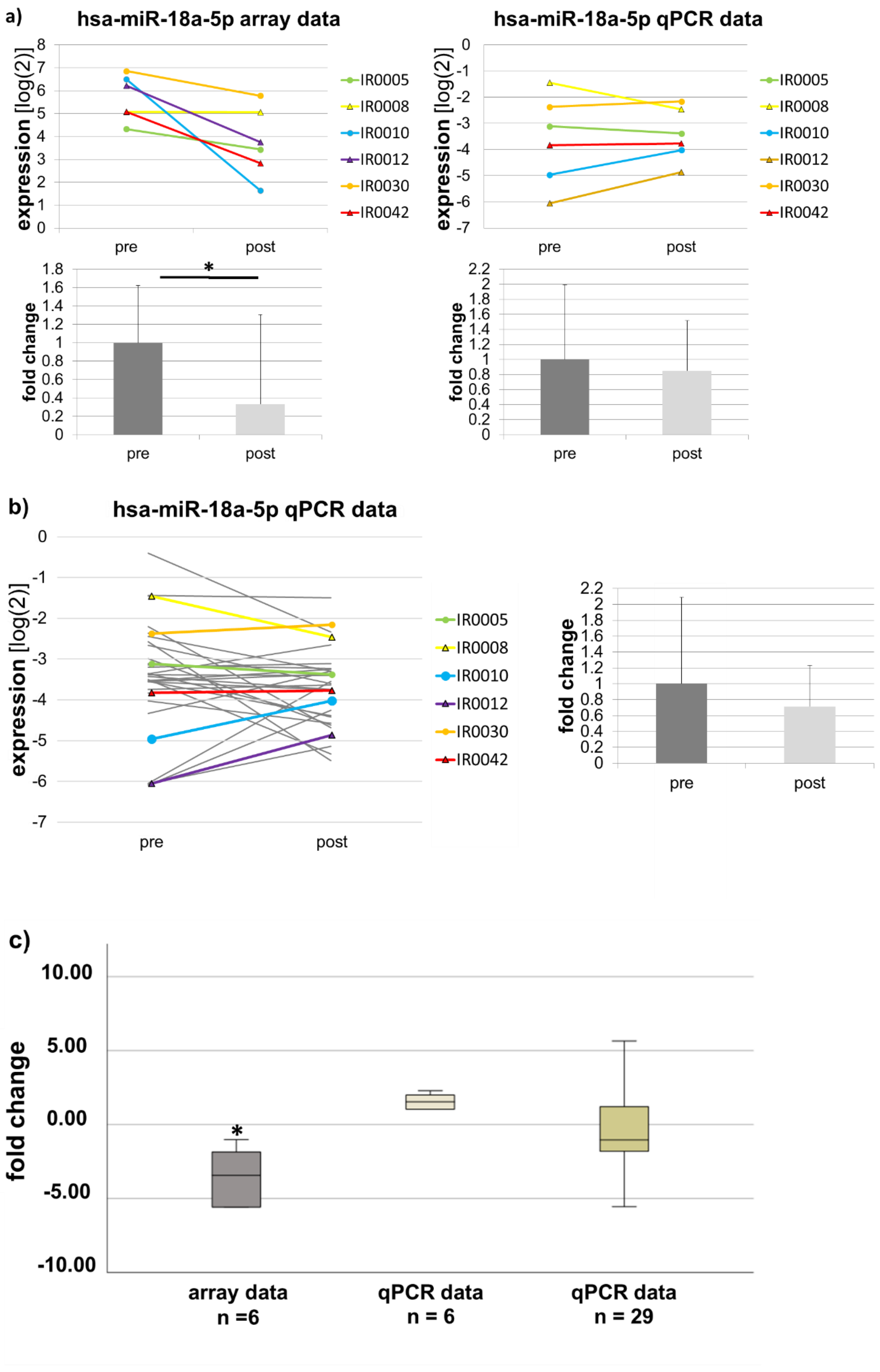
a)
hsa-miR-18a-5p array data
hsa-miR-18a-5p qPCR data
expression [log(2)]
fold change
pre
post
*
IR0005
IR0008
IR0010
IR0012
IR0030
IR0042
b)
hsa-miR-18a-5p qPCR data
expression [log(2)]
fold change
pre
post
IR0005
IR0008
IR0010
IR0012
IR0030
IR0042
c)
fold change
10.00
5.00
0.00
-5.00
-10.00
*
array data
n =6
qPCR data
n = 6
qPCR data
n = 29

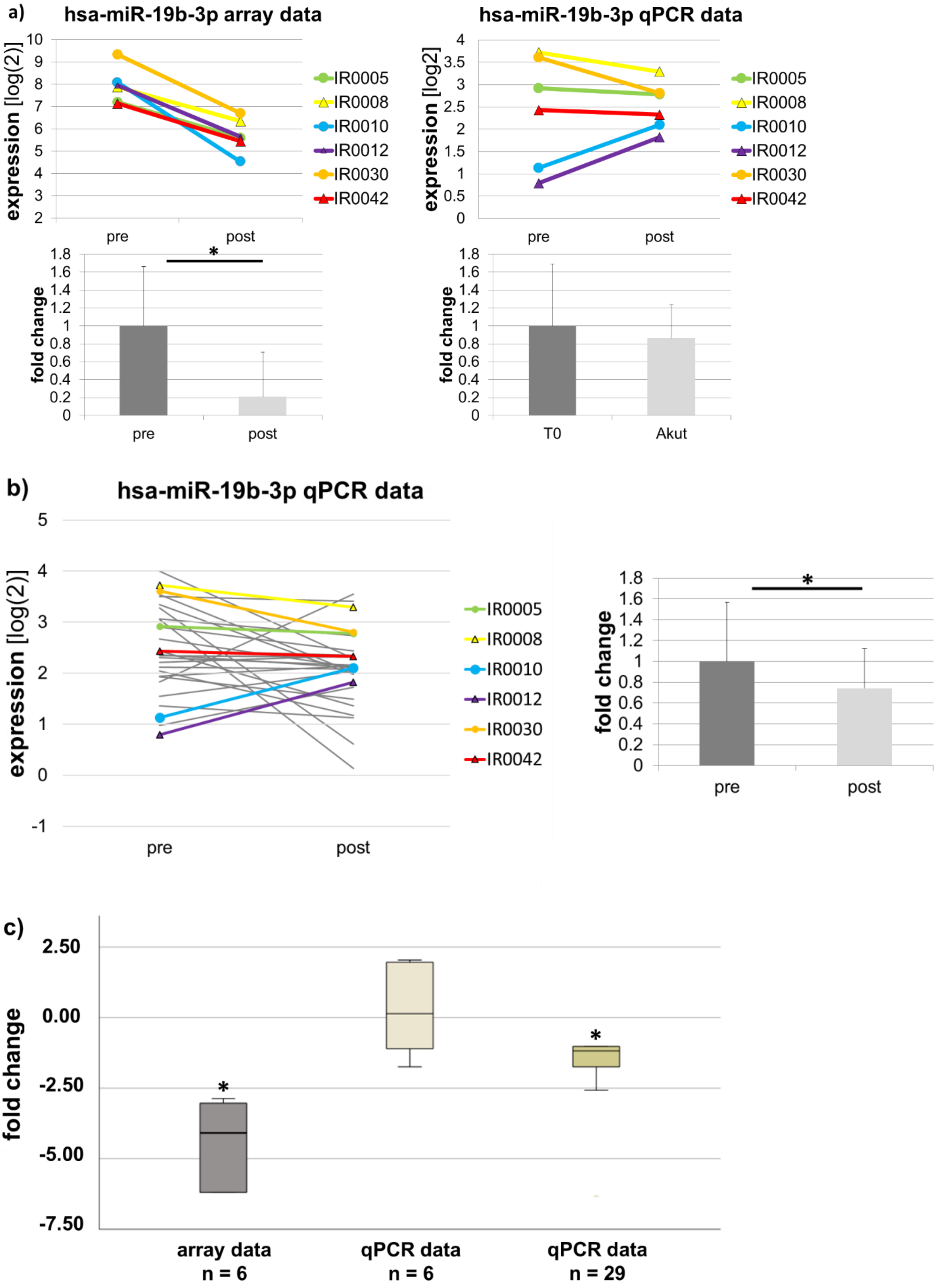
a)
hsa-miR-19b-3p array data
expression [log(2)]
10
9
8
7
6
5
4
3
2
pre
post
IR0005
IR0008
IR0010
IR0012
IR0030
IR0042
hsa-miR-19b-3p qPCR data
expression [log2]
4
3.5
3
2.5
2
1.5
1
0.5
0
pre
post
fold change
1.8
1.6
1.4
1.2
1
0.8
0.6
0.4
0.2
0
*
T0
Akut
b)
hsa-miR-19b-3p qPCR data
expression [log(2)]
5
4
3
2
1
0
-1
c)
fold change
2.50
0.00
-2.50
-5.00
-7.50
array data
n = 6
qPCR data
n = 6
qPCR data
n = 29

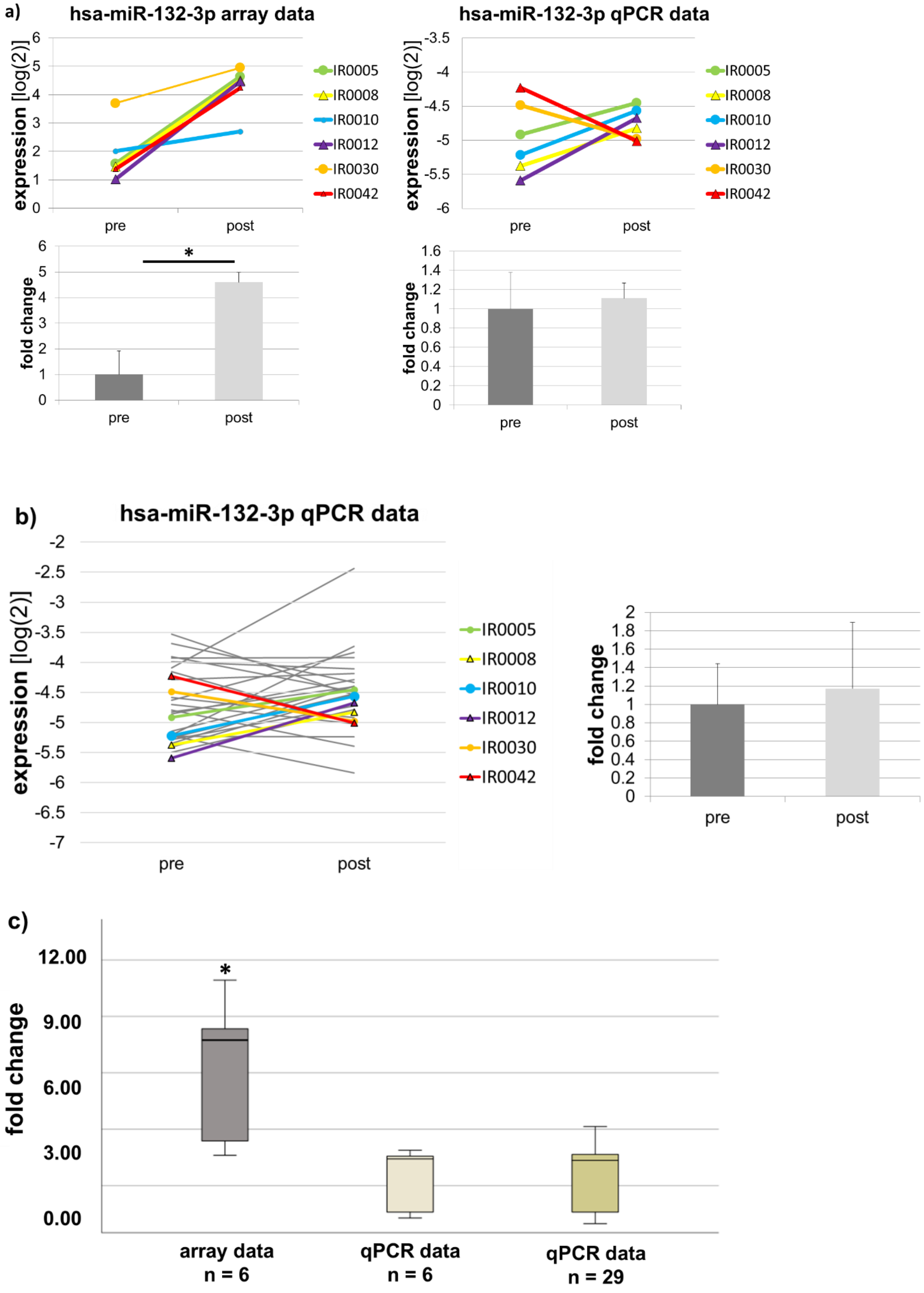
a)
hsa-miR-132-3p array data
expression [log(2)]
pre
post
IR0005
IR0008
IR0010
IR0012
IR0030
IR0042
hsa-miR-132-3p qPCR data
fold change
*
b)
hsa-miR-132-3p qPCR data
c)
fold change
array data
n = 6
qPCR data
n = 6
qPCR data
n = 29

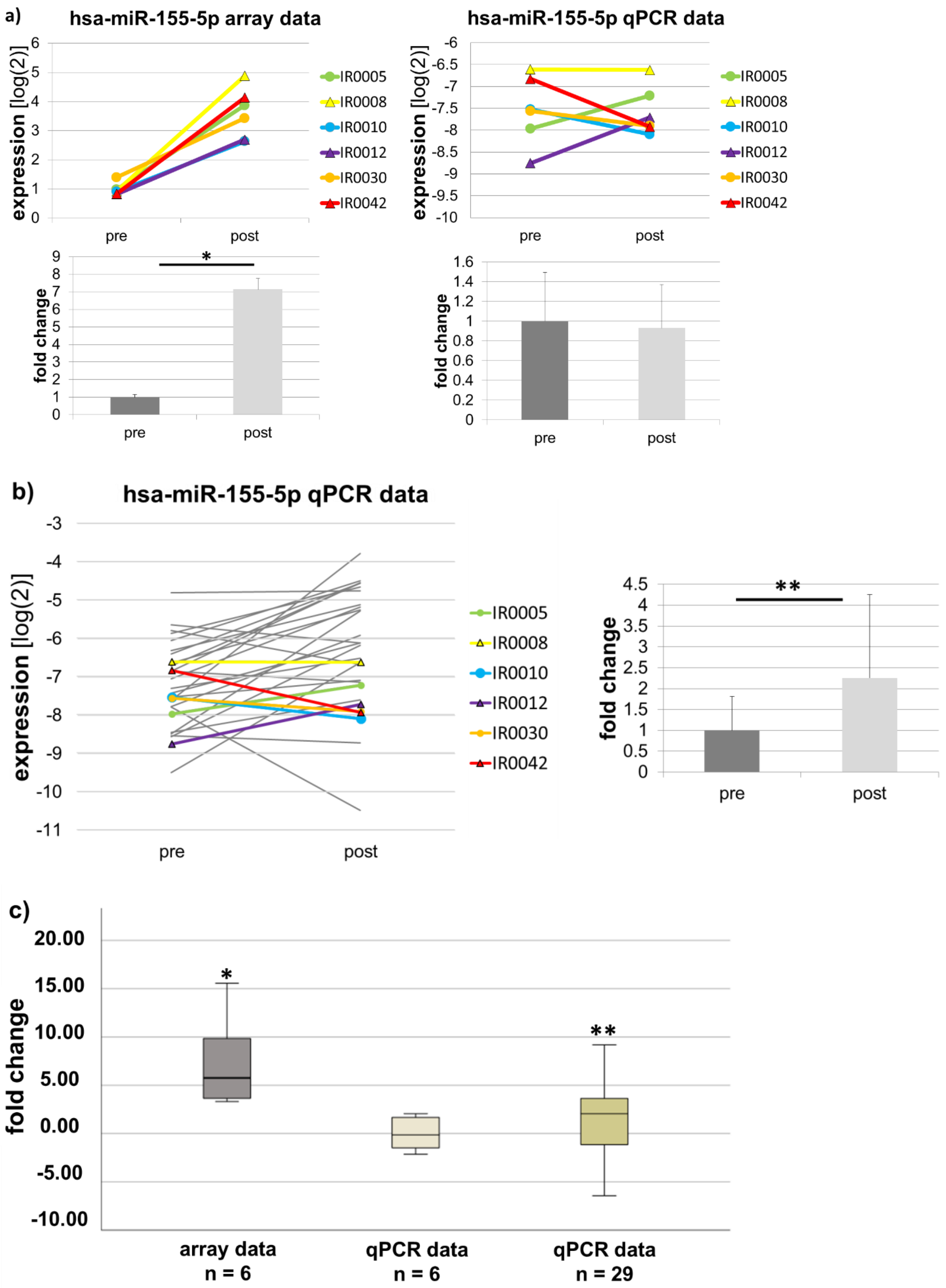

a)
hsa-miR-155-5p array data
expression [log(2)]
pre
post
IR0005
IR0008
IR0010
IR0012
IR0030
IR0042
fold change
*
hsa-miR-155-5p qPCR data
expression [log(2)]
fold change
b)
hsa-miR-155-5p qPCR data
expression [log(2)]
fold change
**
c)
fold change
array data n = 6
qPCR data n = 6
qPCR data n = 29

Suppl. Fig.1 (previous pages). Changes in miR concentrations in skeletal muscle tissue in response to a single bout of exercise. (a) Comparison of microarray and qPCR data (n=6). (b) Changes in miR concentrations as assessed by qPCR in al participants (n=29). (c) Fold changes as detected by microarray (n=6), qPCR (n=6), and qPCR (n=29). Subjects of the MICT-HIIT group are labelled with circles, those of the HIIT-MICT group with triangles.
